# The complete mitochondrial genome sequence of *Schisandra chinensis* (Austrobaileyales: Schisandraceae)

**DOI:** 10.1080/23802359.2019.1638326

**Published:** 2019-07-13

**Authors:** Jeong-Ho Baek, Hyo-Jin Kim, Sang-Ho Kang, Soo-Jin Kwon, Chang-Kug Kim

**Affiliations:** a Gene Engineering Division, Rural Development Administration, National Institute of Agricultural Sciences, Jeonju, Republic of Korea;; b ARES Medicinal Resource Research Institute, Jinan, Republic of Korea;; c Genomics Division, National Institute of Agricultural Sciences, Jeonju, Republic of Korea

**Keywords:** Mitochondrial genome, *Schisandra chinensis*, Schisandraceae family

## Abstract

Chinese magnolia vine (*Schisandra chinensis*) is an economically important oriental medicinal plant that belongs to the Schisandraceae family. The complete mitochondrial genome sequence of *S. chinensis* was 946,141 bp in length. A total of 45 genes was annotated, including 30 protein-coding genes, 12 tRNA genes, and 3 rRNA genes. A phylogenetic tree based on the mitochondrial genome demonstrated that *S. chinensis* was most closely related to *Schisandra sphenanthera* of the Schisandraceae family.

## Main

Chinese magnolia vine (*Schisandra chinensis*), a medicinal plant belonging to the Schisandraceae family, grows in China, Japan, Korea, and Russia and is well-known in both traditional and modern Chinese medicine (Chun et al. 2014). *Schisandra chinensis* has been reported to have several medicinal effects, including for gastrointestinal issues, respiratory failure, insomnia, body fatigue, delaying the aging process, excessive sweating, and cardiovascular protective activity (Szopa et al. 2017).

A sample was obtained from the Jeollabukdo Medicinal Resource Research Institute (voucher number: JA2018-117, geographic coordinate: N 35°46′10″, E 127°22′40″) in Jinan, Korea. Whole genome sequencing was performed using the Illumina Miseq platform (Illumina, CA, USA), the Nanopore GridION platform (Oxford Nanopore Technologies, Oxford Park, UK), and the PacBio RSII platform (Pacific Biosciences, Menlo Park, CA, USA). A total of 7.6 Gb of raw read bases were generated and low-quality bases (<Q20) were trimmed using the NGS QC Toolkit (Patel and Jain 2012). First, initial contigs were generated using a hybrid assembly of Illumina Miseq data and Nanopore sequencing data using the SPAdes assembler (Bankevich et al. 2012). Second, selected contigs were subjected to gap-filling procedures and error-corrected using the CLC Assembly Cell package (QIAGEN Bioinformatics, CA, USA). Finally, genes were annotated using Mitofy and BLAST searches (Alverson et al. 2010).

The complete mitochondrial genome of *S. chinensis* was a circular form 946,141 bp in length. The genome harbored 45 annotated genes, including 30 protein-coding genes, 12 tRNA genes, and 3 rRNA genes. The complete mitochondrion of *S. chinensis* (Accession no. MK860624) was registered to NCBI GenBank.

Genome alignments were performed using MAFFT (Yamada et al. 2016), and a phylogenetic tree was constructed using MEGA 7.0 (Kumar et al. 2016). The phylogeny was generated using 11 reported species, and phylogenetic relationships were analyzed using five common protein-coding genes (*nad3, nad6, nad9, rpl5,* and *rps12*), which exists completely within the initiation and stop codon.

**Figure 1.**
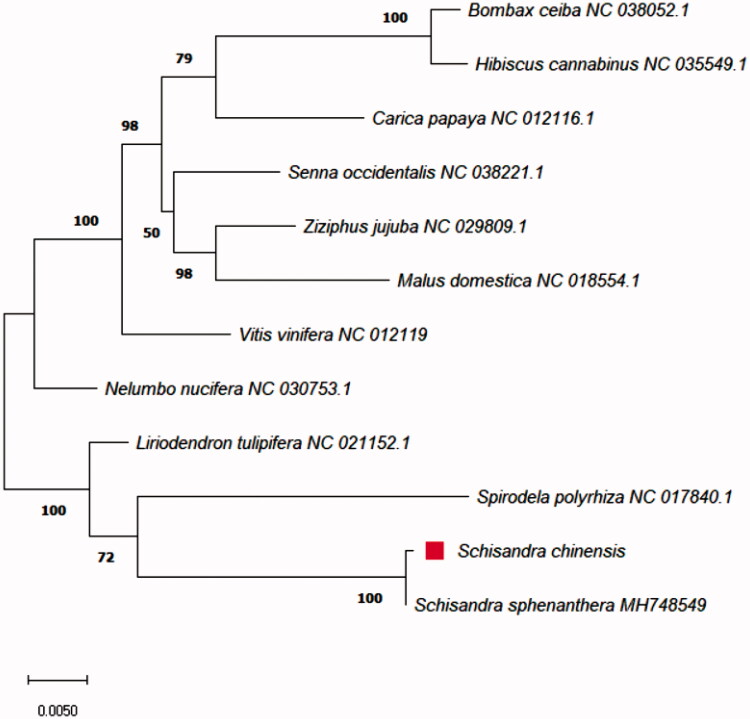
Phylogeny of *S. chinensis* and 11 related species based on their mitochondrial genome sequences. The phylogenetic tree was constructed using the maximum likelihood method with 1000 bootstrap replicates based on five common protein-coding genes from the mitochondrial genomes of 12 species.

Our phylogeny analysis indicated that *S. chinensis* is most closely related to *Schisandra sphenanthera* of the Schisandraceae family (Figure 1). Clinically, *S. chinensis* and *S. sphenanthera* have different pharmacological effects, although there are reports that these two species have common main components (Lu and Chen 2009). This finding is expected to contribute to the development of molecular markers for herbal drugs in the Schisandraceae family.

## Geolocation

The genomic DNA sample used for sequencing was obtained from the Jeollabukdo Medicinal Resource Research Institute (geographic coordinate: N 35°46′10″, E 127°22′40″).

## References

[CIT0001] Alverson AJ, Wei X, Rice DW, Stern DB, Barry K, Palmer JD 2010 Insights into the evolution of mitochondrial genome size from complete sequences of *Citrullus lanatus* and *Cucurbita pepo* (Cucurbitaceae). Mol Biol Evol. 27:1436–1448. 20118192 10.1093/molbev/msq029 PMC2877997

[CIT0002] Bankevich A, Nurk S, Antipov D, Gurevich AA, Dvorkin M, Kulikov AS, Lesin VM, Nikolenko SI, Pham S, Prjibelski AD, et al. 2012 SPAdes: a new genome assembly algorithm and its applications to single-cell sequencing. J Comput Biol. 19:455–477. 22506599 10.1089/cmb.2012.0021 PMC3342519

[CIT0003] Chun JN, Cho M, So I, Jeon JH 2014 The protective effects of *Schisandra chinensis* fruit extract and its lignans against cardiovascular disease: a review of the molecular mechanisms. Fitoterapia. 97:224–233. 24976588 10.1016/j.fitote.2014.06.014

[CIT0004] Kumar S, Stecher G, Tamura K 2016 MEGA7: molecular evolutionary genetics analysis version 7.0 for bigger datasets. Mol Biol Evol. 33:1870–1874. 27004904 10.1093/molbev/msw054 PMC8210823

[CIT0005] Lu Y, Chen DF 2009 Analysis of *Schisandra chinensis* and *Schisandra sphenanthera*. J Chromatogr A. 1216:1980–1990. 18849034 10.1016/j.chroma.2008.09.070

[CIT0006] Patel RK, Jain M 2012 NGS QC Toolkit: a toolkit for quality control of next generation sequencing data. PLOS One. 7:e30619. 22312429 10.1371/journal.pone.0030619 PMC3270013

[CIT0007] Szopa A, Ekiert R, Ekiert H 2017 Current knowledge of *Schisandra chinensis* (Turcz.) Baill. (Chinese magnolia vine) as a medicinal plant species: a review on the bioactive components, pharmacological properties, analytical and biotechnological studies. Phytochem Rev. 16:195–218. 28424569 10.1007/s11101-016-9470-4 PMC5378736

[CIT0008] Yamada KD, Tomii K, Katoh K 2016 Application of the MAFFT sequence alignment program to large data-reexamination of the usefulness of chained guide trees. Bioinformatics. 32:3246–3251. 27378296 10.1093/bioinformatics/btw412 PMC5079479

